# Food Safety Analysis Using Electrochemical Biosensors

**DOI:** 10.3390/foods7090141

**Published:** 2018-09-01

**Authors:** Geetesh Kumar Mishra, Abbas Barfidokht, Farshad Tehrani, Rupesh Kumar Mishra

**Affiliations:** 1 Multiscale Fluid Mechanics Lab, School of Mechanical Engineering, Sungkyunkwan University, Suwon 44-746, Korea; 2 Department of Nano-Engineering, University of California San Diego, La Jolla, CA 92093, USA; abarfi@gmail.com (A.B.); farshad.tehrani@gmail.com (F.T.); 3 Department of Biosciences and Biotechnology, Banasthali University, Rajasthan 304022, India

**Keywords:** food safety, biosensors, electrochemical technique, food analysis

## Abstract

Rapid and precise analytical tools are essential for monitoring food safety and screening of any undesirable contaminants, allergens, or pathogens, which may cause significant health risks upon consumption. Substantial developments in analytical techniques have empowered the analyses and quantitation of these contaminants. However, conventional techniques are limited by delayed analysis times, expensive and laborious sample preparation, and the necessity for highly-trained workers. Therefore, prompt advances in electrochemical biosensors have supported significant gains in quantitative detection and screening of food contaminants and showed incredible potential as a means of defying such limitations. Apart from indicating high specificity towards the target analytes, these biosensors have also addressed the challenge of food industry by providing high analytical accuracy within complex food matrices. Here, we discuss some of the recent advances in this area and analyze the role and contributions made by electrochemical biosensors in the food industry. This article also reviews the key challenges we believe biosensors need to overcome to become the industry standard.

## 1. Introduction

Food safety monitoring is a very important aspect for dealing with threats to human health and well-being of the population. It has emerged as one of the most significant public concerns worldwide due to changing food habits, expansion of mass catering establishments, and the globalization of food supply chains [1]. As food supply chains are becoming global, the need to strengthen food safety and monitoring systems in every country is becoming more evident [2]. Moreover, food quality control is essential for consumer protection as well as for the food industry thus, screening of food contaminants and quantification of food constituents is becoming vital for food industries and consumers [3]. Some strict guidelines are laid out by the regulatory agencies like United States Food and Drug Administration (USFDA) and the European Food Safety Authority (EFSA) which details the maximum levels for certain contaminants in foodstuffs to maintain a high level of human health and consumer protection [4,5]. Presently, a greater part of the food analysis is carried out at the end of the production process using conventional techniques such as chromatography, mass spectrometry, ultraviolet detection, or fluorescence techniques either individually or in combination with other separation techniques [6,7]. These traditional approaches have several limitations. Firstly, as the analysis is carried out at the end of the process, contaminated batches of product can go through an expensive production process before any concerns are raised. Second, these methods of analysis are laborious, expensive, time consuming, require large sample volumes, and highly trained personnel [8]. To overcome these limitations, biosensors offer a possible alternative by allowing screening of food samples for any potential contaminants before the production process was complete.

Biosensors are an example of a new but innovative method to approach important problems in a quality-conscious society and have become powerful tools, especially for food sample analysis [9]. Furthermore, biosensors also offer the possibility of rapid and on-site monitoring, thus providing real-time information in the production process [10]. Among the various reported biosensors, electrochemical biosensors have been very popular and widely used due to their well-understood bio-interaction and detection process [11,12,13,14]. Need of highly sensitive, specific, and rapid analysis, alongside accuracy in analytical measurements has developed significant scope for the development of electrochemical biosensors as a novel detection tool for food commodity [15]. In this regard, the last decade has observed phenomenal growth in the field of electrochemical biosensors for analyses of food and beverages [16,17], detection of genetically modified organisms (GMO) in food [18], etc. However, there are several possibilities yet to be open in these fields for the application of electrochemical biosensors. In this review article we consider recently developed electrochemical biosensors applied for food analysis and safety. We will look up to the aspects of biosensors that present striking substitutes to conventional techniques and instrumentation, briefly explore recent advances in biosensor technologies, and also evaluate their shortcomings. We will finalize the review by putting forward some future ideas and challenges that the electrochemical biosensors have to overcome to establish as the new alternate tool for food analysis and safety.

## 2. Attributes of Electrochemical Biosensor in Food Safety Analysis

In electrochemical biosensors, measurements of signals for biological samples are generally electrochemical in nature, where a bio-electrochemical element serves as the transduction component. Usually in electrochemical biosensors, biological reactions either generate a change in signal for conductance or impedance, measurable current, or charge accumulation which might be measured by conductometric, potentiometric, or amperometric techniques [19]. Investigated reactions are normally detected on a close proximity of an electrode surface and detection techniques are generally chosen based on the electrochemical properties of a specific electrode surface. Electrochemical techniques involve a reference, auxiliary, and a working electrode. Reference electrodes are mostly formed by silver chloride and are kept at a distance from the reaction-site to maintain a stable potential. The sensing electrode acts as a transduction element in the biochemical reaction, whereas a counter electrode sets up contact between the electrolytic solution and electrode surface to apply current to the working electrode [20]. However, in some cases of electrochemical impedance spectroscopy (EIS) based measurements, researchers have also reported two electrode based set-up for detection of various analytes. EIS measurements can be made with a different number of electrodes in different configurations, among which the most common ones are usually called two or three-electrode implementations [21]. One such testimony exploring two electrode-based EIS set-up was reported by Bacher et al., for detection of aflatoxin M1 in milk samples. The change in impedance caused by antigen–antibody interactions was measured at the electrode surface where the first electrode was configured as working electrode (working and sense electrode combined together) and a second electrode was used as a reference electrode (reference and counter electrodes combined together) [22].

Selection of a suitable working electrode is the crucial part for successful electrochemical measurements. Recent years have witnessed several modifications in working electrode materials where various doped or undoped forms of carbon and dimensionally stable anions have been employed, rather than the classic metals such as, mercury, gold, platinum, silver, nickel, and copper [23]. These materials are being used either with some chemical modification or in bare forms for achieving enhanced selectivity, sensitivity, and stability. It has been always a concern about the widespread use of biosensors that they should offer further significant benefits over the existing conventional methods. One such advance is the potential for sensor miniaturization, which results in the sensor requiring greatly reduced sample sizes or volumes. Miniaturization of the working electrodes has opened a new dimension for in vivo or in vitro electrochemical studies with the advancement in micromachining, photolithography, and micro-contact printing [24]. However, demand for a low-cost and disposable electrochemical sensor for easy use in the food industry was recognized with the development of screen-printed electrode-based sensing techniques [25]. These techniques generally involve deposition of electrode materials on inert PVC or ceramic support usually with carbon or noble metals.

**Figure 1:**
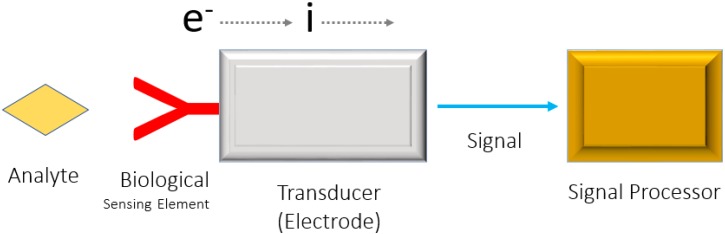
Schematic illustration of mechanism/principle of electrochemical biosensor.

Bioreceptors or biological recognition elements are also an integral part of biosensor system [26,27]. Bioreceptors are biological molecular species, such as antibodies, enzymes, proteins, and nucleic acids that utilize a biochemical mechanism for recognition. Recent research in electrochemical biosensors has focused on bioreceptors since they are very important elements for the specificity of biosensor technologies. A bioreceptor allows the binding between the specific analyte of interest with the sensing surface for measurement with minimum intervention from other components in a complex mixture. Recent development of novel biorecognition molecules, such as synthetic aptamers, DNA, proteins, and viruses has enabled considerable selectivity in food analysis. Moreover, parallel development in the immobilization of bioreceptors through robust attachment methods like electro-deposition and nanoparticle-bound entities at the working electrode interface is a significant step in the improved application of biosensors in food analysis [28,29,30,31,32,33]. Selection of robust and suitable immobilization methodology and precise selection of the bioreceptor molecule plays a great part in the better specificity, selectivity, and affinity for their target analytes in electrochemical biosensing [10]. In food sample analysis, rapid, economical, easy-to-use, and disposable kind of chips are appreciated for electrochemical sensing and several researchers are working in this direction. Figure 1, represents the basic principle involved in electrochemical biosensor detection.

## 3. Challenges Overcome by Electrochemical Biosensors in Food Sample Analysis

Biosensors are being used mainly for three broad categories in food analysis, to control food safety, food quality, and accuracy of analysis [34]. Food sample testing usually focuses on the detection of unwanted contaminants in food, such as biological toxins, antibiotic residues, food allergens, pathogenic microbes, and pesticides. Some of the biosensors are also used to establish or verify the nutritional value of a food product and authenticity analysis seeks to confirm the origin or production process of a food commodity whilst providing information about the adulteration and imitation of the food product [35,36,37]. As indicated by the recently reported literatures, electrochemical biosensors are primarily being explored in food safety rather than quality and validity analysis [38]. Conventional techniques for the detection of mycotoxins or pathogenic microorganisms, pesticides, or antibiotics could only be conducted post-production. This limitation is easily overcome by using the biosensing techniques, which allow food items to be tested at all phases of production including screening of raw materials to the product on the shelf, resulting in more efficient means of ensuring food safety [39]. Furthermore, biosensors also provide improved analysis time in food analysis. By implementing advanced biosensing tools like microarray-based biosensors and microfluidic or lab-on-a-chip platforms, low volume samples can be analyzed directly, thus reducing the need for lengthy, laborious, and expensive sample preparation stages [40]. An important precondition to use conventional techniques in food analysis is the sample homogenization process, which can lead to several problems due to the use of some organic acids and the presence of antimicrobial compounds in many fruits and vegetables. The release of these compounds during sample preparation can hinder the detection of certain contaminants, potentially having harmful impacts on product consumers, a problem not encountered by biosensors as they require little or no sample preparation [41,42].

Rapid analysis is predominantly an additional striking feature of biosensors where toxin accumulation often correlates with time, for instance, fungal toxins are harmful carcinogenic secondary metabolites that affect several food products, including nuts, vegetables, cereals, dried fruit, bread, wine, and meat products. In such instances, biosensors present an attractive alternative and allows reduced detection times, from several days to hours, or even minutes [43]. Additionally, in situ detection facilitates improved portability of analytical tools such as hand-held [44] or mobile devices which usually involves minimal guidance to operate and can also provide the opportunity for integration of real time analysis in food processing set-ups [45].

## 4. Potential Analytes for Food Safety Measurements

Electrochemical biosensors have the great advantage as analytical tools that they can be used for detecting an enormous range of chemical substances. Antibodies or aptamers can be produced for several toxins, bacterial species, pesticides, herbicides, and antibiotic residues, as well as for nontoxic substances, such as proteins and other small molecules for construction of an electrochemical biosensor. These substances can be easily detected using these biosensors as long as they are foreign to the immunizing species [46]. The high affinity of the biorecognition elements for their target analytes allowed the development of very sensitive and specific electrochemical biosensors. These properties comprise the great advantage that has allowed the development and potential use of these biosensors to detect the presence of any desired or undesired chemical or biological entity for ensuring food safety. Among the various food contaminants, pathogenic microbes and mycotoxins are prominently reported in food samples [47,48].

### 4.1. Food Contamination by Pathogenic Microbes

**Figure 2:**
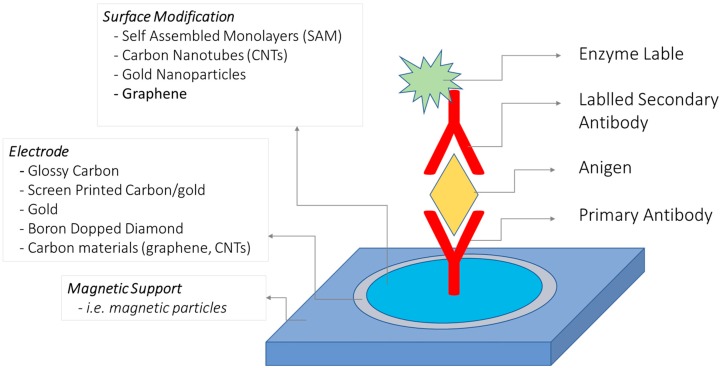
Labeled/nonlabeled antibody based biosensor illustration.

Detection of contaminated food by pathogenic microorganism is an important concern for ensuring food safety, security and public health. Food contamination by pathogenic bacteria like, *Escherichia coli*, *Salmonella typhimurium*, *Staphylococcus aureus*, *Bacillus cereus*, Streptococci, etc., can causes several foods borne illnesses and are responsible for approximately 90% of all food borne diseases. Conventional methods for bacterial identification usually involves various culturing techniques and different biochemical tests which are very time consuming and usually require 2–4 days. Hence, there was a need for adequate monitoring technologies targeting representative pathogenic bacteria at low levels within hours to prevent mortality and morbidity caused by bacterial outbreaks [49]. The effective testing of bacteria requires methods of analysis that meet a number of challenging criteria. Analysis time and sensitivity are the most important limitations related to the usefulness of bacterial testing. An extremely selective detection methodology was also required, because low numbers of pathogenic bacteria are often present in a complex biological environment along with many other nonpathogenic bacteria [50]. Tedious and time consuming detection methods has prompted several groups in the recent years to develop other techniques to reduce the detection time like Polymerase Chain Reaction (PCR) and Enzyme Linked Immunosorbent Assay (ELISA). However, both techniques have limitations that exclude their widespread implementation. These limitations include accurate primer designing, requirement of specific labeled secondary antibody, and their failure to distinguish spore viability [51,52,53,54]. They are easy to use, require minimal reagents, and can be deployed in fields. Among the reported biosensors, electrochemical biosensor has emerged as sensitive technique for bacterial detection due to multiple advantages, such as fast response, low cost, and capability of miniaturization. Moreover, the time for detection using electrochemical biosensors methods has been cropping as methods improve and some newer methods take as little as 10 min. Impedance-based biosensors are particularly attractive for food safety analysis since they allow label-free detection with high sensitivity [55]. Figure 2 represents the schematic of labeled/label free detection of analytes using biosensor approach.

Recently, numerous electrochemical biosensors have been reported using impedimetric, potentiometric, and voltammetric techniques, for the detection of several bacteria and parasites. The achievement of sensitive detection limits for microbes was supported by incorporating nanomaterials like gold nanoparticles (AuNPs), carbon nanotubes (CNTs), and graphene oxide (GO), or by amplification of enzyme-labeled probes. Recently a method was reported for detection of the most common food pathogen, *Salmonella typhimurium*, in pork, exploring AuNPs and GO using an EIS-based technique with a limit of detection (LOD) of 3 CFU/mL [56]. In an additional work, detection of *Staphylococcus aureus* was reported exploring single-walled carbon nanotubes (SWCNT) and a potentiometric technique achieving 800 CFU/mL in pig skin [57]. In addition to these detection methods, some recently reported methods have explored various nanomaterials for food sample analysis. Lately, Izadi et al., established an electrochemical DNA-based biosensor for *Bacillus cereus* in milk and infant formula. They explored AuNPs to prepare a modified pencil graphite electrode that could detect Bacillus cereus as low as 10^0^ CFU/mL [58]. For the detection of *Salmonella* spp., Ma et al. have developed an impedimetric biosensor based on specific recognition of the bacteria by a specific aptamer. Detection range for the developed biosensor was reported between 2.4 and 2.4 × 10^3^ CFU mL^−1^ and a LOD of 3 CFU mL^−1^ was achieved. Recovery studies were performed in pork samples by spiking 10 to 1000 CFU mL^−1^ of bacteria and recoveries were close to 100% [56]. In another research work, a DNA biosensor for the detection of pathogenic bacteria *Aeromonas hydrophila* in fish and vegetables was developed based on the electrochemical technique [59]. An impedimetric biosensor was demonstrated for the rapid detection of *Salmonella typhimurium* based on the poly [pyrrole-co-3-carboxyl-pyrrole] copolymer-supported aptamer in a spiked food sample [60]. Other electrochemical biosensing platforms have also been reported for multiplexed determination of pathogenic bacteria. For instance, a bio-bar coded genosensor based on Au NPs and MNPs in a screen-printed carbon electrode chip was proposed by Zhang et al. [61]. The biosensor was able to detect *Salmonella enteritis* as low as 0.5 ng mL^−1^ and 50 pg mL^−1^ of *Bacillus anthracis* in 2.5 h. In a similar way, Dou et al. [62] developed an immunosensor for *Escherichia coli* O157:H7 (*E. coli* O157:H7) detection using carbon screen-printed low density arrays coated with MWCNTs/sodium alginate/carboxymethyl chitosan composite films and further functionalized with HRP labeled antibodies. LOD for *E. coli* O157:H7 detection was reported as 3.47 × 10^3^ CFU mL^−1^.

### 4.2. Food Contamination by Toxins

**Figure 3:**
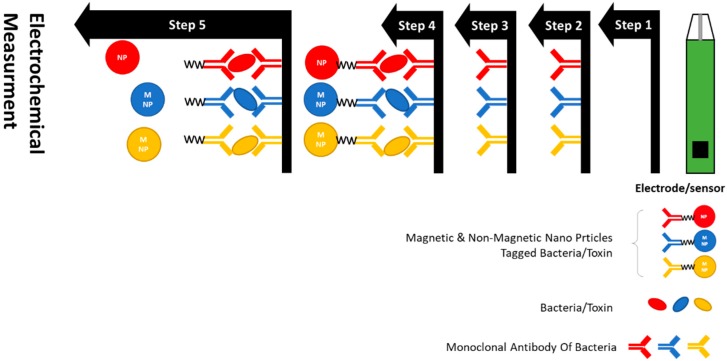
Schematics for bacteria and toxin detection using antibody based electrochemical biosensor.

Food toxins are usual constituents covering a large variety of molecules, generated by the metabolism of fungi, algae, plants, or bacteria with harmful effects on human health. Fungal toxins, also known as mycotoxins, are chemically heterogeneous toxic secondary metabolites of fungal origin with a broad range of toxic effects. More than 400 different mycotoxins have been presently recognized with a large variety of chemical structures, physicochemical, and toxicological properties. Some mycotoxins persuade genetic disorders and progressions of carcinogenesis, others have teratogenic, embryotoxic, and allergenic effects [63]. Contamination of agricultural crops by mycotoxins can occur at various levels of food chain, for instance, preharvest, harvest, drying, storage, or transportation. The toxicity of these molecules has directed many countries to setup strict regulations for their control in food and feed and the consequent establishment of legislation to control their possible contamination. In the European Union, the maximum allowed concentrations of ochratoxin A (OTA) are in the 0.5–80 µg kg^−1^ range, depending on the kind of food, while aflatoxin B1 (AFB1) is regulated in the 0.1–12 µg kg^−1^ range [64]. Detection of mycotoxin is challenging as these molecules are present in low concentrations in complex matrices, and they may occur in various combinations produced by a single or by several fungal species. Several mycotoxins have been noticed in food samples during recent past; among them, aflatoxins and OTA were studied extensively and detection of mycotoxins using electrochemical biosensors has remained an active research area. A simple and sensitive biosensor was recently developed for the detection of OTA via covalent immobilization of OTA aptamers on screen-printed electrodes (SPE). The authors reported a linear detection range for OTA between 0.15 and 2.5 ng/mL exploring impedimetric technique [65]. In another work, Catanante et al. developed a folding mechanism-based electrochemical biosensor for OTA using MB-tagged anti-OTA aptamers. In this work, they established various aptamer coupling strategies using, diazonium polyethylene glycol, and HMDA coupling. The best performance was recorded through oxidation of amines using HMDA on SPCE with LOD of 0.01 ng/mL [66]. In an additional work, the authors reported the detection of AFM1 in raw milk using covalent immobilization of a hexa-ethylene glycol-modified aptamer on SPE using impedimetric biosensor. They reported a reproducible detection range between 2 and 150 ng/L. Additionally, authors have also analyzed spiked milk samples for AFM1 and the obtained results correlated well with other established commercial methods [67]. Several other electrochemical biosensors have also been reported harnessing the selectivity of antibodies or combining the selectivity and stability of aptamers for OTA detection [68]. Various strategies have been proposed for signal enhancement using AuNPs, MNPs, and graphene derivatives. An additional approach combining AuNPs and polymers for an efficient detection of OTA was reported by Etvugyn et al. [69]. In this work, an impedimetric aptasensor was prepared by drop casting a suspension of Au NPs stabilized by the dendrimeric polymer Botlorn H30^®^ (Perstorp Speciality Chemicals AB, Perstorp Sweden) onto gold electrodes previously modified by electropolymerized Neutral Red. Thiolated aptamer specific to OTA was covalently attached to Au NPs via Au-S bonding. The interaction of the aptamer with OTA induced the conformational switch of the aptamer from linear to guanine quadruplex form followed by consolidation of the surface layer and an increase of the charge transfer resistance. The aptasensor enabled OTA detection in the 0.1 to 100 nM range in the presence of at least 50-fold excess of ochratoxin B and an LOD of 8 pg mL^−1^ was achieved. The applicability of the aptasensor for real sample assay was confirmed by testing spiked beer samples. For AFM1detection, diverse approaches have been reported recently. Dinckaya et al. [70] developed an aptamer-based electrochemical biosensor by modifying gold electrodes by a cysteamine SAM, grafting on AuNPs followed by a specific binding of thiolated aptamer to AFM1, layer-by-layer. The AFM1 binding event provided a linear response between 1 and 14 ng mL^−1^, with a LOD of 1 ng mL^−1^ by EIS detection. The biosensor was successively applied to a real milk sample, with recoveries close to 100%. A representative scheme for detection of bacteria/toxin exploring various bioreceptors and nanoparticles is presented as Figure 3.

## 5. Conclusions and Future Directions

Biosensors have been established as striking analytical devices for fast screening of food impurities, hazardous chemicals, and toxins for food safety. Electrochemical biosensors offer numerous benefits, in particular easy miniaturization and probable integration with multiplexed analysis using easy-to-use formats. Over the recent past, a variety of new immobilization supports have been proposed for making self-assemblies, biomolecule integration, and labels or signal enhancers by exploring various nanoparticles or carbon-based nanostructures to produce electrochemical biosensors of improved analytical performance. The recent trends in the development of such electrochemical sensors for real-time, on-site monitoring have proficiently determined the time restrictions involved in conventional laboratory methods by providing high-throughput, multi-analyte, and portable biosensors. Although biosensors display clear advantages over traditional methods, a perfect biosensing technique does not yet exist and there are many difficulties in its development to be overcome. Currently, many biosensors could not easily have used for on-site monitoring, therefore, very few are currently available commercially. Transforming biosensor technology into a commercial product from lab scale research is still hampered by some major shortcomings. Foremost, the burden of the demand for the development of sensitive biological sensing layers has pushed researchers to design highly complex and expensive methods which ultimately end up an extremely costly component. The stability of the biological receptors immobilized within these complex structures is sometimes not maintained in real sample analysis conditions, which constitutes an uncertain block preventing biosensor commercialization. Research activities in the field of artificial receptors such as aptamers have increased in the last few years, but only a few of them have been produced for a limited number of target analytes. More efforts have to be done in this field. Nevertheless, it is almost expected that the future of electrochemical biosensors will involve partnership with information communications technology to assist food producers, retailers, authorities, and even consumers, in their decision-making process by equipping them with the necessary tools. The combination of different types of biosensors or hybrid biosensors has great promise; for instance, fusion of electronic tongues with electronic noses may further increase the identification capabilities of such a biomimetic system, specifically, the biological system. The advantage of real-time monitoring in food manufacture, particularly of dairy and brewed products, further improves the effectiveness of biosensors and pushes their commercially availability to general public. The prospect of biosensing techniques and authenticity in sample analysis at all levels of the food supply chain will motivate the widespread usage of electrochemical biosensors and will repute as a food productions and food safety tool of the future.
